# Resurgence of Minimal Stimulation *In Vitro* Fertilization with A Protocol Consisting of Gonadotropin Releasing Hormone-Agonist Trigger and Vitrified-Thawed Embryo Transfer

**DOI:** 10.22074/ijfs.2016.4903

**Published:** 2016-06-01

**Authors:** John Zhang

**Affiliations:** Reproductive Endocrinology and Infertility, New Hope Fertility Center, New York, United States

**Keywords:** *In Vitro* Fertilization, Clomid, GnRH Agonist

## Abstract

Minimal stimulation *in vitro* fertilization (mini-IVF) consists of a gentle controlled ovarian stimulation that aims to produce a maximum of five to six oocytes. There is a misbelief that mini-IVF severely compromises pregnancy and live birth rates. An appraisal of the literature pertaining to studies on mini-IVF protocols was performed. The advantages of minimal stimulation protocols are reported here with a focus on the use of clomiphene citrate (CC), gonadotropin releasing hormone (GnRH) ago- nist trigger for oocyte maturation, and freeze-all embryo strategy. Literature review and the author’s own center data suggest that minimal ovarian stimulation protocols with GnRH agonist trigger and freeze-all embryo strategy along with single embryo transfer produce a reasonable clinical pregnancy and live birth rates in both good and poor responders. Additionally, mini-IVF offers numerous advantages such as: i. Reduction in cost and stress with fewer office visits, needle sticks, and ultrasounds, and ii. Reduction in the incidence of ovarian hyperstimulation syndrome (OHSS). Mini-IVF is re-emerging as a solution for some of the problems associated with conventional IVF, such as OHSS, cost, and patient discomfort.

## Introduction

The widespread increase in the daily dosage of gonadotropins was introduced in *in vitro* fertilization (IVF) protocols in the late 1980s and early 1990s. Some of the reasons for this increase include: i. High doses of gonadotropins increased the number of oocytes retrieved in good and poor responders, and ii. It allowed the formation of more embryos providing excess embryos for cryopreservation (1,2). The introduction of gonadotropin-releasing hormone (GnRH) agonists and antagonists as a suppression for the premature luteinizing hormone (LH) surge/ ovulation further allowed clinicians to use higher doses of gonadotropins (3,4). Although there is no doubt that the high oocyte yield in conventional IVF contributed to better success rates, it has resulted in several drawbacks such as: i. High treatment cost (5), ii. Increased incidence of multiple pregnancies when more than one embryo is transferred (6), and iii. Increased risk of the potentially life-threatening ovarian hyperstimulation syndrome (OHSS) when human chorionic gonadotropins (hCG) is used for final oocyte maturation (7). The conventional longstimulation protocol uses GnRH agonists for suppression of the anterior pituitary thus preventing the LH surge (4). The long GnRH agonist stimulation protocol became accepted as the standard protocol in many countries. GnRH agonist is usually started in the mid-luteal phase of the preceding menstrual cycle followed by stimulation with high doses of gonadotropins leading to multifollicular recruitment (8). However, the GnRH agonist protocol has many side effects including longer duration of treatment, formation of ovarian cysts and symptoms of estrogen deprivation (mood changes, hot flushes, and headaches) (9). Additionally, some of the known side effects of conventional IVF include the intake of several daily injections by the patient what causes pain, frustration, and skin/muscle irritation. These side effects, along with the exponential improvement in the embryology field with its associated better implantation rates, have led many to question the continued need for aggressive stimulation and to encourage revisiting minimal stimulation protocols (10,12). Minimal stimulation consists of a gentle controlled ovarian stimulation that produces a maximum of five to six oocytes (13). Minimal stimulation IVF (mini-IVF) is re-emerging as a solution for some of the problems associated with conventional IVF. The purpose of this report is to revisit the advantages of minimal stimulation protocols over conventional IVF with a focus on a protocol that uses GnRH agonist trigger rather than hCG for oocyte maturation and that utilizes a freeze-all embryo strategy rather than fresh embryo transfer. 

## Materials and Methods

A literature review of clinical prospective and retrospective available studies in PubMed for relevant publications in English through January 2015 was performed. In addition to IVF, the following search terms were used: conventional, mini-IVF, mild ovarian stimulation, clomiphene citrate (CC), freeze-all embryo, and GnRH agonist trigger. In addition, references from all relevant articles were reviewed. Titles and abstracts of all citations identified in the search were reviewed. The full-text article was retrieved if the citation was potentially relevant to miniIVF. 

## Results

### Minimal stimulation in vitro fertilization protocol

**Fig.1:**
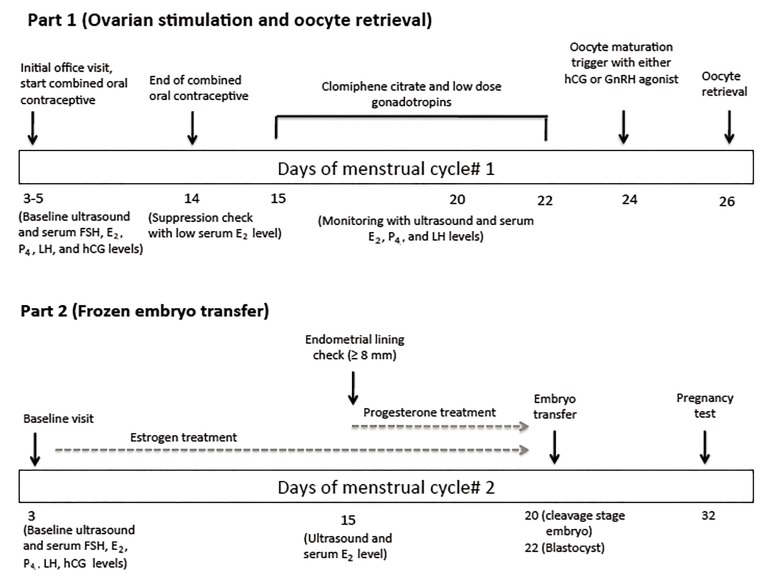
A schematic diagram of the mini-IVF protocol (part 1) with freeze-all embryo transfer (part 2) used at our center. E_2_; Estradiol, P4; Progesterone, FSH; Follicle-stimulating hormone, LH; Luteinizing hormone, hCG; Human chorionic gonadotropin, GnRH; Gonadotropin releasing hormone and mini-IVF protocol; Minimal stimulation *in vitro* fertilization protocol.

After oral contraceptive pill pre-treatment for 14 days, adequate suppression is usually confirmed with a low estradiol (E_2_) level of <75 pg/mL. Minimal ovarian stimulation is started with an extended regimen (from day 3 of the cycle until the day before triggering) of CC (50-100 mg/day orally) in conjunction with gonadotropin injections starting on days 4-7 of the cycle with 75-150 IU of human menopausal gonadotropins (hMG) daily. Patients usually receive both CC and low dose gonadotropins, and the dose given depends on the ovarian reserve status and the body mass index (BMI) of the patient. The final maturation of oocytes is usually induced by either intramuscular hCG or GnRH agonist (intramuscularly or nasally) (Fig .1). When patients desire fresh embryo transfer, hCG is the preferable method for oocyte maturation. Zarek and Muasher (13) have reported a protocol in 31 patients where they used 100 mg of oral CC on days 3 to 7 of the cycle followed by 150 IU of gonadotropins daily started on day 8 of the cycle. They have used GnRH antagonist (0.25 mg of ganirelix acetate) daily for LH surge suppression and 10,000 IU of hCG for final oocyte maturation trigger. In this study, the mean number of mature oocytes retrieved was 4.2, the mean number of embryos transferred was 2.4, and the clinical pregnancy rate per cycle was 42%. Williams et al. (14) compared mini-IVF protocol, using sequential protocol of CC (100 mg of orally on days 3 to 7) and gonadotropins (150 IU of gonadotropin daily starting on day 9) ± GnRH antagonist to suppress the LH surge (n=50 participants), to conventional IVF protocol, using the standard long GnRH agonist protocol (n=52 participants). Despite the fact that mini-IVF yielded significantly lower number of oocytes compared to the conventional stimulation IVF protocol (3.7 vs. 13.1, respectively, P<0.05), both protocols produced similar pregnancy rates. 

At our institution (New Hope Fertility Center, USA) retrieved oocytes are fertilized and subsequently cultured until the cleavage stage or preferably the blastocyst stage. Figure 1 represents a summary diagram that summarizes a protocol that has been used at our institution over the last five years and used by Kato et al. (15) and Teramoto and Kato (16) in Japan. This protocol uses mini-IVF with GnRH agonist trigger and freeze-all embryo strategy, as suggested by previously published literature (14,15,17,19). Good quality blastocysts are vitrified and a single thawed blastocyst is typically transferred in a subsequent artificially prepared frozen embryo cycle. At our center, 564 infertile women (age<39) undergoing their first IVF cycle between February 2009 and August 2013 were randomly allocated to either mini-IVF with single embryo transfer as seen in Figure 1 (n=285) or conventional IVF (n=279) with double embryo transfer (20). The primary outcome was cumulative live birth rate per woman over a 6-month period. We found that the cumulative live birth rate was 49% (140/285) for mini-IVF and 63% (176/279) for conventional IVF [relative risk (RR): 0.76; 95% confidence interval (CI): 0.64-0.89]. There were no cases of OHSS after mini-IVF compared to 16 (5.7%) moderate/severe OHSS cases after conventional IVF. >Gonadotropin consumption was significantly lower with mini-IVF compared to conventional IVF (459 ± 131 vs. 2079 ± 389 IU, P<0.0001) (20). 

### Advantages of using clomiphene citrate in miniin vitro fertilization

CC has traditionally been used as the most fundamental drug for ovulation induction in the treatment of infertility worldwide (21). Chemically, CC is a nonsteroidal triphenylethylene derivative that exhibits both estrogenic agonist and antagonist properties (14,15,17,19). Approximately, 85% of CC dose is eliminated from the blood after 6 days, although traces may remain in the circulation for months. CC is a mixture of 2 geometric isomers, enclomiphene and zuclomiphene, in a 3:2 ratio. Enclomiphene is the more potent isomer that is primarily responsible for ovulation induction. Zuclomiphene is the less-active isomer and cleared far more slowly from the blood (21,22). At the cellular level, CC binds nuclear estradiol receptor (ER) for a long period of time thus depleting ER concentration by slowing down and ultimately depleting ER replenishment (22). In ovulation induction, CC depletes ER at the level of the hypothalamus thus suppressing the usual negative feedback by circulating E_2_(22,23). This triggers the hypothalamus to secrete high levels of GnRH secretion that will stimulate endogenous release of follicle-stimulating hormone (FSH) and LH by the pituitary (23). The increase in serum gonadotropins will then stimulate follicular activity at the level of the ovary. In usual ovulation induction, CC is used for 5 days; a period of time that increases both endogenous LH and FSH (22). On the contrary, when CC is administered for more than 5 days, LH release decreases. Thus, clinicians around the world started using CC, instead of GnRH agonists or antagonists, as a suppressive agent for premature LH surge in many IVF protocols, especially mini-IVF (15,16). Messinis and Templeton (24) reported that prolonged administration of CC inhibited positive feedback and thus prevented the LH surge. In that study, they demonstrated that CC administration for 15 days (days 2 to 16 of the cycle) produced a continuous and progressive increase of basal LH levels with no LH surge and no ovulation. 

### Trigger with nasal gonadotropin-releasing hormone agonist

Conventional IVF typically uses an intramuscular or a subcutaneous injection of hCG at 500010,000 IU. It is well known that hCG can trigger OHSS, especially in high-risk groups such as women with polycystic ovary syndrome (PCOS), low BMI, young, and good responders (25). Rather than using hCG, protocols described in the miniIVF and conventional IVF literature have used nasal administration of GnRH agonist as the maturation trigger (15,16,26,27). The reasoning behind using GnRH agonist is first to avoid OHSS, and second to have a natural maturing process of oocytes based on the endogenous LH/FSH surge thus maintaining a natural luteal function (28). GnRH agonist trigger have been shown to be beneficial in situations like repeated IVF failure, empty follicle syndrome and repeated retrieval of immature oocytes, with the hypothesis that some patients may require the FSH surge, in addition to the LH surge, to promote final oocyte maturation resembling the natural midcycle surge of gonadotropins (29,30). Several studies have reported that cycles where GnRH agonist was used as the maturation trigger produced comparable number of mature oocytes to cycles where hCG was used (31,32). Griesinger et al. (33) demonstrated that the use of GnRH agonist to trigger final oocyte maturation yields is a comparable number of mature oocytes and comparable embryonic development to that achieved with hCG trigger. Interestingly, Humaidan et al. (34) demonstrated that GnRH agonist trigger produced higher number of metaphase II (MII) oocytes than hCG trigger. However, in that same study, GnRH agonist trigger as a final oocyte maturation was associated with a lower pregnancy rate, a lower live birth rate, and a higher rate of early miscarriage (28). It seems more likely that GnRH agonist induces a luteal phase defect. This luteal phase defect may result from the short half-life of the induced LH surge, leading to premature luteolysis of corpus luteum and significantly lower steroidal and non-steroidal hormones, thus affecting endometrial receptivity (35). However, these evaluations become less relevant in protocols where a freeze-all embryo strategy, rather than transferring embryos in the same fresh cycle, is employed. Finally, GnRH agonist can be administered nasally making it a patient-friendly drug by avoiding the injectable hCG (15,16). 

### Freeze-all embryo strategy

The general success rates for frozen-thawed embryo transfers have increased in the past few years. The Centers for Disease Control and Prevention (CDC) collected data on assisted reproductive technology (ART) success rates of all American fertility clinics from 1997 to 2011. According to those data, success rates of both fresh and frozen-thawed embryo transfer cycles (donor eggs not included) have increased over the past 14 years for women of all ages. Interestingly, the increase in success rates seems to be greater for frozen-thawed embryos compared with fresh embryos (36). Furthermore, data from children born from frozen-thawed embryo transfer cycles show fewer perinatal complications of preterm birth, small for gestational age, low birth weight, and perinatal mortality compared with children born from fresh embryo transfers (37,39). Additionally, outcomes of singletons born after frozen-thawed embryo transfer seem more similar to naturally conceived singletons (40). So far, the etiology of these differences is unknown, although suboptimal endometrial development has been suggested to be a risk factor for the adverse outcomes of ART (41). Proper placentation with fresh embryo transfer may be jeopardized by the supraphysiological concentrations of estrogen and progesterone (P ^4^), leading to worse perinatal outcomes compared with frozen-thawed embryo transfers in a more neutral physiological environment. There seems to be a less receptive endometrium in cycles with ovarian stimulation, and children born from the transfer of frozen-thawed embryos had better outcome compared with children born from cycles with fresh embryo transfer. Several recent studies have shown that the success rates of frozen-thawed embryo transfer are similar, if not better, to the success rates of fresh embryo transfer (42,43). Whether frozen-thawed embryo transfers should be performed in natural or in artificial cycles is not clear. Several studies compared frozen-thawed embryo transfers in natural or artificial cycles and found no differences in pregnancy rates or live birth rates (44,45). 

## Conclusion

There is still resistance to use of minimal stimulation protocols because of the fear of having few oocytes. There is also a misbelief that minimal stimulation severely compromises pregnancy and live birth rates. It is clear that IVF treatment is stressful and costly to lots of patients. Multiple office visits including injections, blood draws, and ultrasounds can add to the stress. Additionally, stress could emanate from the high cost of IVF medications and procedures, as well as IVF outcomes that include complications of multiple pregnancies (such as preterm birth, preeclampsia, cesarean section, etc.). Although severe OHSS is rare, it constitutes a real threat for high-risk patients. Interestingly, there is a lack of literature pertaining to the outcome of mini-IVF in obese older women, especially those with diminished ovarian reserve. We have recently shown that female adiposity might impair oocyte number and maturity in conventional IVF but not in minimal stimulation IVF, suggesting that gentle ovarian stimulation might yield healthier oocytes in obese women. Thus, future studies are needed to address the optimal IVF protocol in this patient population. There is no doubt that minimal stimulation protocols is disadvantageous to oocyte donation and elective oocyte cryopreservation, and that it limits the number of available embryos. However, molecular differences at the level of the oocyte seem to be real between conventional IVF versus mini-IVF. Additionally, molecular differences between frozen-thawed embryos and freshly cultured human embryos have been reported. Interestingly, success rates after frozen-thawed embryo transfer are now nearing the success rates of fresh embryo transfer supporting the hypothesis of so called freeze-all embryo strategies in IVF to optimize success rates. Finally, there is a critical need for high quality randomized controlled trials to determine which cryopreservation protocol is best and whether a freeze-all embryo strategy is justified in IVF treatments. 
